# Mouse anatomy ontologies: enhancements and tools for exploring and integrating biomedical data

**DOI:** 10.1007/s00335-015-9584-9

**Published:** 2015-07-25

**Authors:** Terry F. Hayamizu, Richard A. Baldock, Martin Ringwald

**Affiliations:** The Jackson Laboratory, 600 Main Street, Bar Harbor, ME 04609 USA; MRC Human Genetics Unit, IGMM, University of Edinburgh, Crewe Road, Edinburgh, EH4 2XU UK

## Abstract

Mouse anatomy ontologies provide standard nomenclature for describing normal and mutant mouse anatomy, and are essential for the description and integration of data directly related to anatomy such as gene expression patterns. Building on our previous work on anatomical ontologies for the embryonic and adult mouse, we have recently developed a new and substantially revised anatomical ontology covering all life stages of the mouse. Anatomical terms are organized in complex hierarchies enabling multiple relationships between terms. Tissue classification as well as partonomic, developmental, and other types of relationships can be represented. Hierarchies for specific developmental stages can also be derived. The ontology forms the core of the eMouse Atlas Project (EMAP) and is used extensively for annotating and integrating gene expression patterns and other data by the Gene Expression Database (GXD), the eMouse Atlas of Gene Expression (EMAGE) and other database resources. Here we illustrate the evolution of the developmental and adult mouse anatomical ontologies toward one combined system. We report on recent ontology enhancements, describe the current status, and discuss future plans for mouse anatomy ontology development and application in integrating data resources.

## Introduction

Anatomy is an integral component for many types of biological data, including gene expression patterns, mutant and disease phenotypes, and normal and pathological processes. Databases serve an important role in capturing and storing diverse types of data from different sources, thus facilitating data integration and analysis. Due to differences in experimental scope and in the collection and reporting of results, authors describe anatomy-related data in different ways in terms of nomenclature and levels of tissue resolution. Anatomical ontologies aim to overcome semantic and granularity differences, and to enhance data representation, by providing standardized vocabularies in which anatomical terms are connected to other terms in meaningful ways. The ontologies also provide a framework to represent additional knowledge about the anatomy, including spatial organization, tissue and organ system classification, as well as temporal and developmental lineage information.

Anatomical ontologies for the mouse have been proven essential for standardized description of gene expression and other mouse data related to anatomy. The Gene Expression Database (GXD; www.informatics.jax.org/expression.shtml; Smith et al. 2015-this issue) uses mouse anatomy ontology terms for annotating many types of developmental expression data, both from the published literature and from direct submissions. The eMouse Atlas Project (EMAP; www.emouseatlas.org/emap/home.html; Davidson et al. 1997) uses 2D and 3D spatial models of embryos to provide gross anatomical and histological representations of mouse development. These models serve as the framework for collecting and digitally storing spatial patterns of gene expression by the eMouse Atlas of Gene Expression (EMAGE; www.emouseatlas.org/emage/home.php; Richardson et al. 2014). As an integral component of these and other mouse data resources, the anatomy ontology enables consistent identification of mouse anatomical structures and standardized textual descriptions of anatomy-related information. It also serves as a means for making the data accessible for aggregation and analysis, as well as further integration via the anatomy.

Recently, the anatomy ontology for the mouse has undergone extensive changes, with regards to both the content of anatomical terms and the structural organization of the ontology itself. In the following sections, we present an overview of the evolution and current status of the mouse anatomy ontologies, including some of the rationale for ontology content augmentation, restructuring of the hierarchies, and other enhancements. We also discuss future plans for anatomy ontology development and application in integration with other data resources.

## An anatomy ontology for mouse development: early versions

**Fig. 1:**
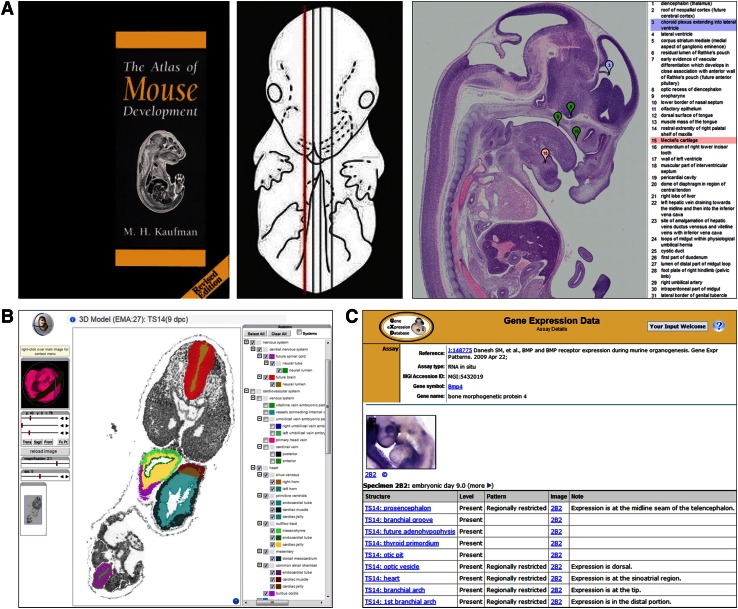
Mouse anatomy ontologies enable standardized description of mouse anatomy for data from different sources. **a** Histological sections from The Atlas of Mouse Development, with anatomical structures identified by Kaufman, provided the initial list of tissues for the developmental mouse anatomy ontology. Ontology terms have since been used to label structures included in an online version of this resource. **b** Ontology terms have also been used by the eMouse Atlas to identify anatomical domains in 2D and 3D models of mouse embryos. **c** GXD uses mouse anatomy ontology terms for textual description of gene expression patterns. Consistent use of a common standardized anatomy nomenclature enables and facilitates data integration between resources. Cover image A reprinted from Kaufman (1994) with permission from Elsevier

The anatomical ontology for the developing mouse originated as a Tissue Index for the second edition of *The Atlas of Mouse Development* (Kaufman 1994). The initial list comprised terms representing structures identified in serial histological sections of mice throughout the course of embryonic development (Fig. 1a). Term labels were based on generally accepted names for anatomical structures, with synonyms included as appropriate. Anatomical terms were grouped by stage and, subsequently, by organ system (Bard et al. 1998; Kaufman and Bard 1999).

The original ontology contained about 8000 anatomical terms, with each term representing a distinguishable anatomical entity at a specific developmental Theiler stage (TS; Theiler, 1989). The stage-specific anatomy terms were organized as sets of simple uniparental hierarchical trees. The intent was to describe each stage-specified embryo as being progressively divided into non-overlapping named parts, with one of the objectives to label anatomical domains within the 2D and 3D eMouse Atlas (EMA; Fig. 1b). Thus, the initial anatomy hierarchies utilized exclusively “part of” relationships indicating, e.g., where a structure is located, or what higher order structure or system it is a subdivision of. For instance, the heart has subparts: atria and ventricles, as well as endocardial and muscular tissue components. Parts of organ systems are also represented; for example, the heart and vascular system are components of the cardiovascular system. Overall, ontology terms were divided into 26 separate hierarchies, one for each of the developmental stages, from TS1 through TS26.

This version of the ontology was used extensively for the annotation and integration of mouse expression results by GXD (Fig. 1c) and other resources. While very useful for this purpose, limitations of the ontology also became clear. Probably most notable were the limitations imposed by the tree structure, allowing anatomical terms to have only one hierarchical parent. For example, “brain” could only be represented as part of the “nervous system” but not as part of the “head,” and searches for expression data in “head” would not return data for “brain.” Another issue was the eventual need to provide anatomical terms for the postembryonic stages, including the adult mouse.

## An anatomy ontology for the postnatal mouse

The necessity to also represent the anatomy for the adult mouse and the need for an improved ontology representation led to the development of the Adult Mouse Anatomy (MA) ontology (Hayamizu et al. 2005) which currently includes about 3300 terms. With the eventual goal to provide a unified representation for all mouse anatomy, our strategy was to follow the basic framework of the developmental anatomy, while expanding the scope of the ontology to structures found only postnatally, and also addressing concerns regarding the hierarchical structure and relationships, and ontology format.

From its inception, the postnatal mouse anatomy ontology was structured as a directed acyclic graph (DAG) in which a term can be represented as a child of more than one hierarchical parent, enabling alternative views of the anatomy. Furthermore, the MA was organized both as a partonomy, in which a term can be a component “part of” its parent (e.g., the brain is a regional part of the head and also a component of the central nervous system) and as what is known as a subsumption classification, in which a term “is a” subclass of its parent (e.g., the brain is an organ; the head is a body region).

Terms from the MA have been used to annotate many different types of data pertinent to adult and other postnatal mouse anatomy. Owing to its utility for resources dealing primarily with MA, the plan is to maintain the MA as a separate ontology. However, the objective from the outset has been to eventually merge and harmonize the developmental and adult versions of the mouse anatomy ontology. The improved ontology representation of the MA served as an important template for reorganizing the developmental ontology and extending it to include postnatal anatomy as well.

## Building a new ontology for mouse development: EMAPA and EMAPS

**Fig. 2:**
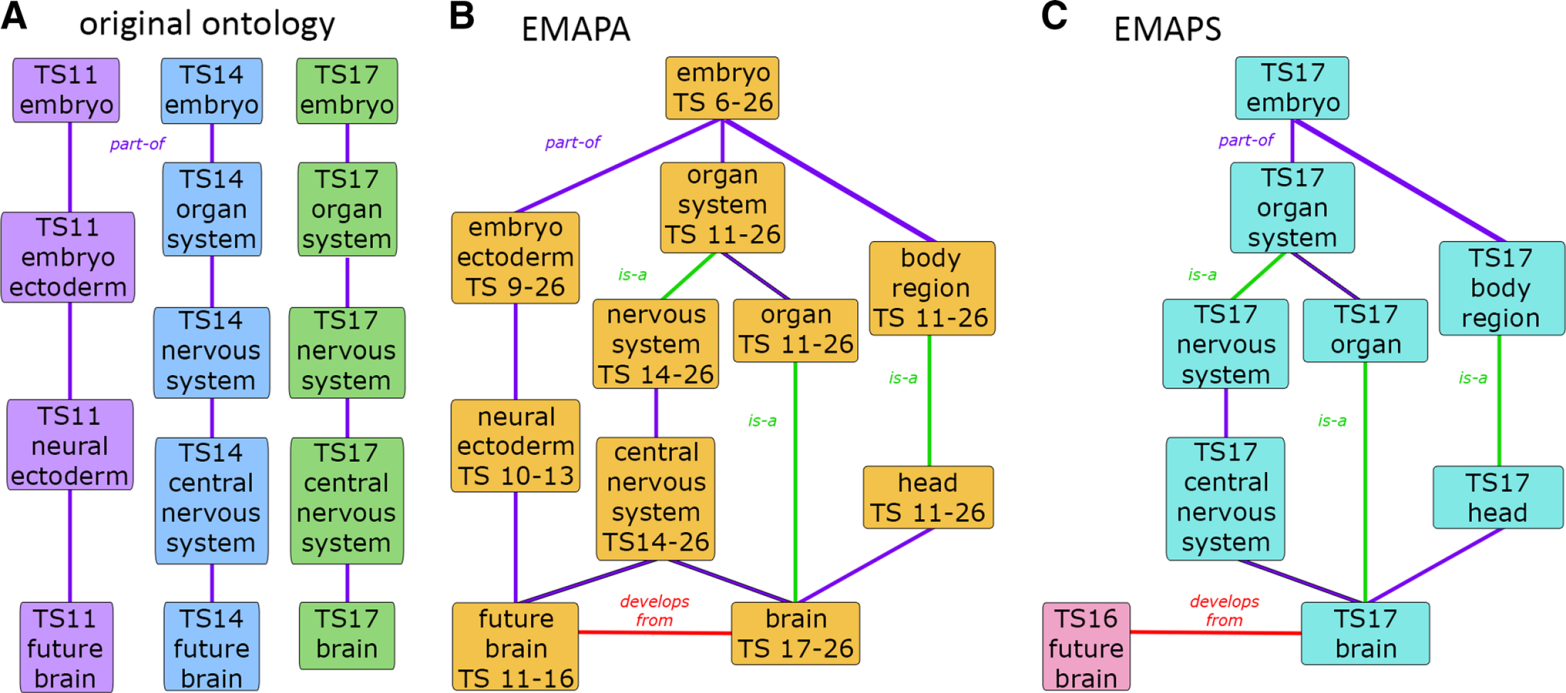
Anatomy ontologies present anatomical terms within a hierarchically organized format, and describe relationships between the anatomical structures represented. **a** The original anatomy ontology for the developing mouse was designed as a simple uniparental hierarchy, exclusively using part-of relationships, with separate trees for the different Theiler stages of development. **b** The EMAPA ontology now provides a unified representation of mouse anatomy for all stages of development, and supports multiple parentage and relationship types. **c** Stage-specific EMAPS hierarchies containing only those terms relevant for a specific stage are derived from the EMAPA using stage-range information for each term contained within the EMAPA file

As presented above, the early mouse anatomy ontology comprised stage-specific anatomical terms divided into 26 separate hierarchies for different stages (Fig. 2a). Multiple separate hierarchies can be difficult to deal with. Such an ontology system is often inefficient with regards to managing terms and relationships, and for maintaining consistency.

In order to address these and other ontological issues, a non-timed “abstract” representation of the mouse anatomy, referred to as EMAPA, has been developed in which an anatomical structure is represented as a single-unique term. An EMAPA term represents a specific structure during its entire existence, has a unique name and unique numerical identifier (i.e., EMAPA id). The ontology file includes specific information pertaining to the range of stages at which the anatomical structure is considered to be present (“start_at” and “ends_at” stages) for each term. Furthermore, all EMAPA terms are contained and organized within a single ontology (Fig. 2b), covering all stages from conception to adulthood. The EMAPA anatomy is now considered to be the primary anatomy ontology for mouse development from which timed versions of the ontology can be derived, and distributed as such through the OBO Foundry Resource (www.obofoundry.org).

Stage-specific terms, designated EMAPS, are derived automatically by GXD based on information contained in the EMAPA ontology file. EMAPS id numbers are constructed to indicate both the EMAPA parent term and the relevant TS. All EMAPS terms for a given stage can be organized into a stage-specific EMAPS hierarchy (Fig. 2c). GXD will continue to annotate expression results to stage-specific instances of anatomical structures (EMAPS terms). Timed versions of the ontology will continue to be made available to those requiring these data, for example resources that use or point to GXD’s expression data.

The new ontology system is much easier to manage because only the EMAPA version needs to be maintained and refined. Editorial procedures have been developed that allow effective co-curation of the ontology between GXD and EMAP in order to incorporate additional terms and other refinements driven by data curation as well as other information sources.

## Expanding the new anatomy ontology for mouse development

The developmental anatomy ontology has undergone substantial expansion and refinement (Hayamizu et al. 2013). Overall, extensions have been predominantly driven by requirements for annotating gene expression data, from both published literature and from large-scale mouse gene expression projects, by GXD and EMAGE. The GenitoUrinary Molecular Anatomy Project (GUDMAP; Little et al. 2007) has contributed extensively to the urinary and reproductive system sections of the ontology. Additional terms have been added in response to input from the Mammalian Phenotype Ontology (MP; Smith et al. 2005) and Uberon Anatomy Ontology (Mungall et al. 2012) and, more recently, the 3D Mouse Limb Anatomy Atlas (DeLaurier et al. 2008) and Molecular Atlas of Lung Development (LungMAP) groups. Information from additional published resources as well as from domain experts is used to validate terms as well as to appropriately integrate them within the ontology. This ontology is a community resource and the ontology editorial group welcomes suggestions for extensions and amendments to refine the concepts and add detail in systems not covered in great depth.

The entire EMAPA ontology has now been extended through newborn (TS27) and postnatal (TS28) stages of mouse anatomy, with the latter substantially augmented by terms and relationships from the MA ontology. As of July 2015, the EMAPA ontology contains nearly 6300 EMAPA terms, resulting in more than 28,500 derived EMAPS terms. Efforts are underway to fully harmonize the EMAPA and MA representations of anatomy for the postnatal mouse, and cross-references (‘xref’s) to TS28 EMAPS terms have been added to the MA ontology file (available at the OBO Foundry).

## Alternative views of mouse anatomy

The initial EMAPA was still represented as a uniparental hierarchy, using “part of” relationships exclusively. To support multiple parentage, as discussed for the MA, the simple tree (uniparental DAG) structure of the EMAPA was converted to a more general DAG (see Fig. 2b). This hierarchical structure enables any anatomical term to have more than one parent term, and also supports the inclusion of other types of relationships relevant to anatomy, such as the “is a” relationship (more on this below).

The stage-specific nature of the original ontology trees meant that the hierarchies could be modeled differently at specific stages of development. For example, as shown in Fig. 2a, “future brain” was modeled as a subterm of “neural ectoderm” at TS11–13, but was considered to be a part of the central nervous system at TS14–16. Since the initial “abstract” ontology allowed a term to have only one parent, separate “future brain” EMAPA terms had to be created for each of the distinct stage ranges, generating unwanted redundancies. Conversion of the developmental ontology to a multi-parental DAG format (see Fig. 2b) meant that all relevant relationships could be included for a single term, obviating the need for redundant terms. Term names have been revised where appropriate and terms were merged where determined to be redundant. In the case of merges, all term labels and numerical identifiers have been retained in the ontology file, as primary term labels or synonyms, and as 1° and 2° (alternate) ids.

The EMAPA has been considerably augmented with the inclusion of an extensive subsumption classification (that is, describing subclasses via “is a” relationships). For example, all terms for the various epithelia, included as a subterm for a majority of structures in the mouse, have been represented in a single tree under the parent class “epithelium.” Many existing “part_of” relationships were determined to be more appropriately modeled as “is a” relationships. As additional classification terms have been identified, term content has been extensively expanded as well. Portions of the hierarchy have also been substantially reorganized in order to appropriately integrate new terms and revised relationships. Furthermore, many classes and relationships at the top-most levels of the hierarchy have been simplified and reorganized in order to improve clarity, and to provide a more accurate and complete representation of the anatomy.

## Navigating the mouse anatomy ontology

The EMAPA and MA ontologies are available from the OBO Foundry resource (www.obofoundry.org) in both Open Biomedical Ontologies (OBO) and Web Ontology Language (OWL) file formats, and can be viewed using widely available ontology editing tools, such as OBO edit (oboedit.org) and Protegé (protege.stanford.edu). Furthermore, online resources providing interfaces for viewing the ontologies include Ontobee (www.ontobee.org) and the Ontology Lookup Service (OLS; www.ebi.ac.uk/ontology-lookup/), as well as the GXD and eMouse Atlas websites. These resources offer different sets of tools for searching and navigating the ontologies, useful for different applications. Here we further describe access to the new mouse developmental ontology and the various functionalities provided by the GXD resource in greater detail.

**Fig. 3:**
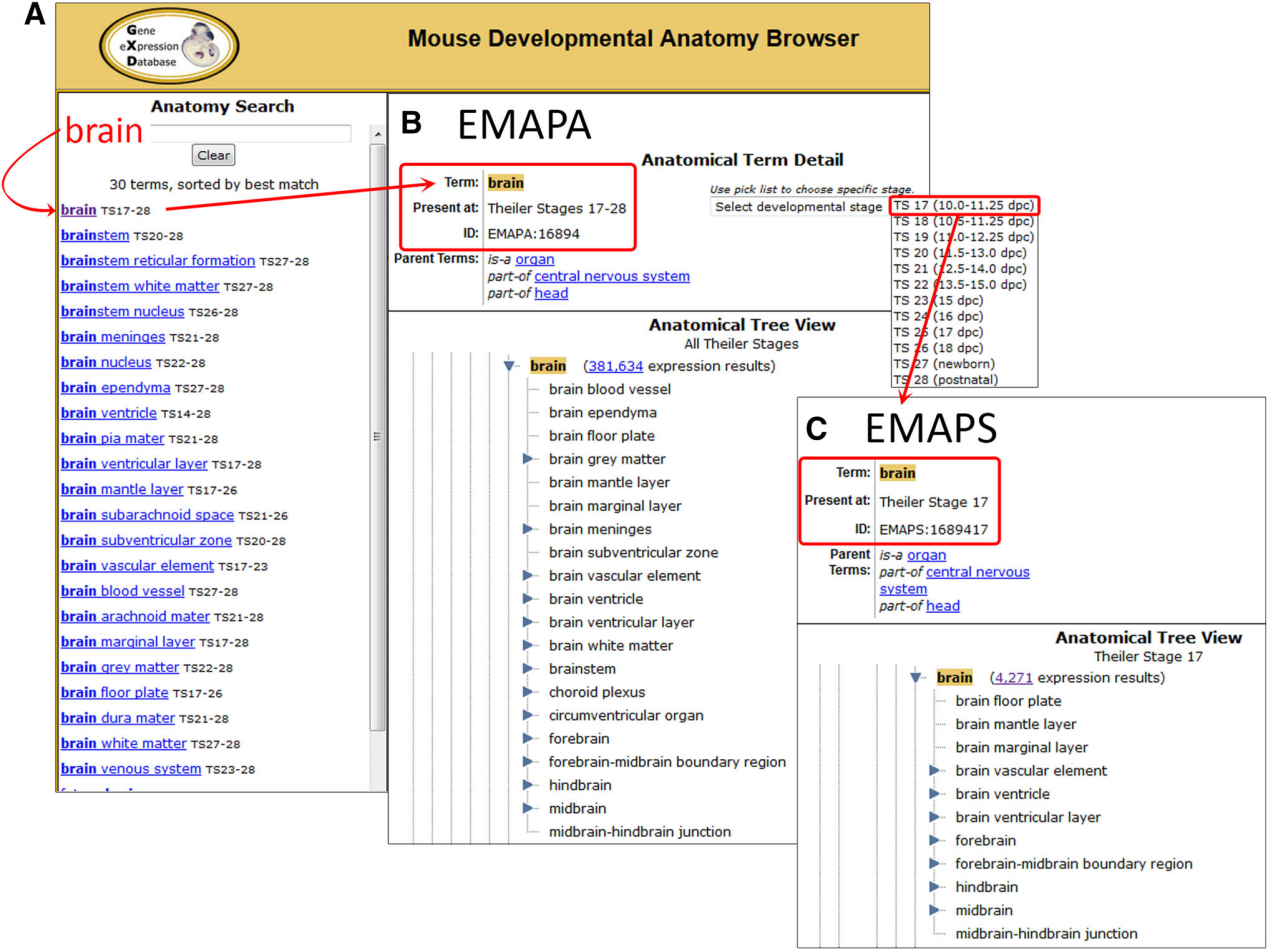
The Mouse Developmental Anatomy Browser enables text-based searches as well as tree-based browsing of the ontology. **a** The Anatomy Search tool enables searching for anatomy terms using structure names and synonyms. **b** and **c** Anatomical Term Detail and hierarchical Tree Views are displayed for both “abstract” EMAPA (**b**) and stage-specific EMAPS (**c**) representations of the anatomy ontology, with the ability to freely toggle between them. Direct links are provided to GXD expression result summaries for the selected term and its hierarchical children

The Mouse Developmental Anatomy Browser (www.informatics.jax.org/vocab/gxd/anatomy/) enables access to both EMAPA and EMAPS versions of the anatomy. Using the Anatomy Search function (Fig. 3a), one can initiate a word search for one or more character strings, which returns a list of terms and synonyms matching your search, and then select a specific EMAPA term. The selected term is then displayed in the Anatomical Term Detail section (Fig. 3b), which provides additional information for each term such as its ID number, the range of stages during which the corresponding anatomical structure is present, and all relevant parent terms and relationships. The anatomical term is also highlighted in an Anatomical Tree View showing the entire hierarchy for the EMAPA. Within the Tree View, the EMAPA hierarchy can be explored interactively by scrolling and by expanding and collapsing sections, and new terms can be selected. A drop-down list in the Term Detail section allows selection of specific developmental stages, and permits the user to toggle between non-timed EMAPA and staged EMAPS terms (Fig. 3c), and views of the corresponding abstract and stage-specific hierarchies. Tree Views for both EMAPA and EMAPS versions of the ontology also provide access to associated gene expression data in GXD (see below).

## Integration with mouse Atlas resources

Recently, an online interface has been developed to provide high-resolution digitalized images of the original histological sections from Kaufman’s *The Atlas of Mouse Development* (www.emouseatlas.org/emap/eHistology/). Pursuant to its origin as the Tissue Index for the *Atlas,* terms from the anatomy ontology have been used to annotate these plates, with links to the eMouse Atlas, EMAGE and GXD, where structures are also labeled with mouse developmental anatomy ontology terms. The eMouse Anatomy Atlas (EMA) portal also hosts an interactive anatomy ontology viewer where stage-specific anatomical terms are linked to anatomical domains in a range of 2D and 3D representations of mouse embryos for each TS throughout mouse development, as well as to associated gene expression data stored by the EMAGE resource.

Together with the spatial representation of corresponding anatomical domains in EMA and EMAGE, the anatomy ontology will serve as an important data integration hub. Integration and interactivity for exploring data across multiple resources are critical for biomedical researchers wanting to access data from resources such as EMA/EMAGE and GXD and the anatomy ontology is a key foundation for this process.

## Gene expression data analysis and integration

**Fig. 4:**
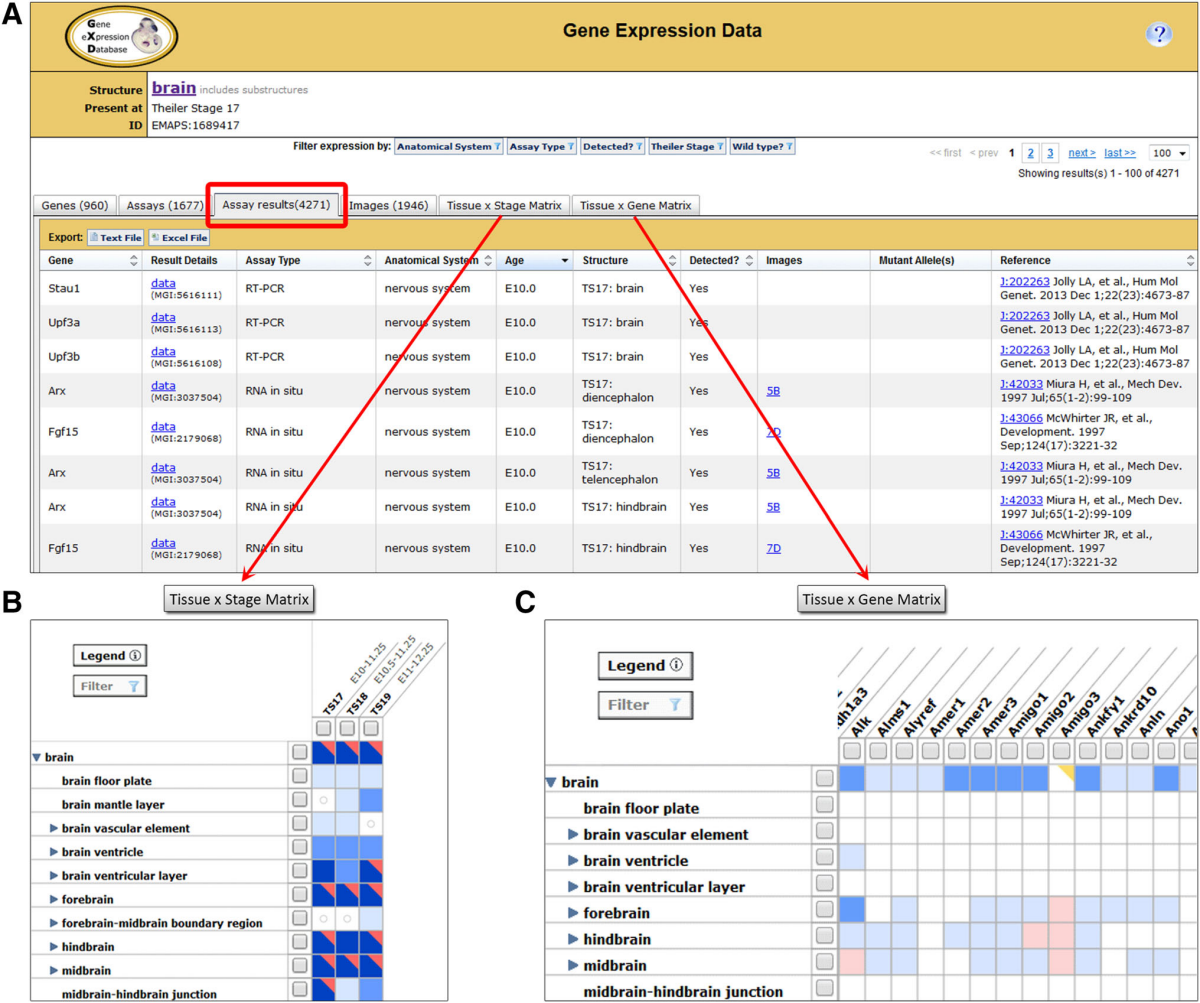
GXD results summaries provide separate tabs for Genes, Assays, Assay Results, Images, and matrix views which present expression data at different levels of detail. **a** The Assay Results tab provides a report of all annotated results relevant to the selected anatomical structure and/or other query parameters, and features links to corresponding Assay Detail pages. **b** The Tissue x Stage Matrix presents a combined spatial and temporal overview for a set of expression results. **c** The Tissue × Gene Matrix enables a comparison of expression patterns for all genes in a results set. Summaries can be iteratively refined by modifying the search or by applying various data filtering options

For each selected term in the GXD Mouse Anatomy Tree View (Fig. 3), the number of associated GXD expression results is indicated, together with a link to a summary of all expression results that have been annotated by GXD to the corresponding anatomical structure or its substructures. Expression data links from the “abstract” view of the hierarchy (from EMAPA terms: Fig. 3b) lead to summaries that show the expression data for all developmental stages at which the selected anatomical structures occur (Fig. 4a). Links from a stage-specific (EMAPS; Fig. 3c) view provide access to the expression data for the selected anatomical structure at the selected specific TS.

GXD currently has almost 1.5 million annotated expression results, covering all hierarchical levels of the anatomy and all developmental stages. Expression results are annotated to about 11,000 stage-specific EMAPS terms, which are derived from nearly 3500 EMAPA terms, thus covering 55 % of all EMAPA terms.

GXD has recently introduced two types of matrix-structured views enabling visualization of gene expression results in the context of both non-timed EMAPA and stage-specific EMAPS hierarchical views of the anatomy ontology. The tissue-by-developmental stage matrix (Fig. 4b) provides a high-level overview of spatio-temporal patterns of expression. For the tissue-by-gene matrix (Fig. 4c), expression for multiple genes is displayed concurrently. Both types of matrices can be expanded and collapsed along the hierarchically organized anatomic structure axis. Anatomy-based matrix views provide intuitive and interactive summaries of GXD results from which users can navigate to pages with more detailed data (see also Finger et al. 2015).

As mentioned above, anatomical structures in the eMouse Atlas are labeled with mouse developmental anatomy ontology terms. EMAGE also uses the anatomy ontology to complement spatial annotations of gene expression data with standardized textual annotations. A new anatomical section browser is being built that will enable users to interactively explore, select labeled anatomical domains, and look up pertinent expression data in EMAGE and GXD. Other projects and resources using the mouse developmental anatomy ontology terms to record expression data include EurExpress (Diez-Roux et al. 2011) and GUDMAP. This has facilitated the integration of expression data from these projects into EMAGE and GXD.

## Other anatomy-based data integration

Many types of biological data relate to anatomy. Using the same anatomical terms to describe the anatomy enables the data to be correlated and integrated. Within the Mouse Genome Informatics (MGI) resource, GXD and the Cre Portal (www.creportal.org) use the same stage-specified mouse anatomy terms to describe both endogenous gene patterns for wild type and mutant mice, and in situ reporter expression patterns for knock-in and transgenic mice expressing Cre recombinase. The Mouse Genome Database (MGD; Eppig et al. 2015) uses the Mammalian Phenotype Ontology (MP; Smith et al. 2005) to describe abnormal mutant phenotypes for the mouse. Many MP terms relate to anatomical entities. Over 4400 MP terms have been associated with EMAPA and MA terms (initially directly, as described by Gkoutos et al. 2005, and more recently indirectly through Uberon terms, see below), thus allowing for anatomical integration and correlation of phenotype and expression data. Mouse anatomy ontology terms are also being used to specify anatomical locations, e.g., for biological processes, as part of the Gene Ontology (GO) project (Gene Ontology Consortium 2010) at MGI. There are currently over 10,700 GO terms that include cross-references to mouse anatomy terms, including close to 2400 distinct stage-specific anatomy terms.

Data integration based on anatomy is also being pursued for data from different species in order to enable comparative analysis. MA and EMAPA have contributed to Uberon, a cross-species anatomy ontology (Mungall et al. 2012). Currently, Uberon includes over 14,200 terms overall. 3072 of these terms correspond to, and include cross-references to, MA terms, and 3549 of these correspond to, and include cross-references to, EMAPA terms. Thus, 95 % of all MA terms and 56 % of all EMAPA terms are currently represented in, and cross-referenced by, Uberon. For example, the EMAPA term ‘limb bud’ (EMAPA:35944) is represented as a cross-reference (‘xref’ in OBO format) to the Uberon class “limb bud’ (UBERON:0004347). Uberon is incorporating similar cross-references to anatomical ontologies from other species, such as Drosophila (Costa et al. 2013), zebrafish (Van Slyke et al. 2014), Xenopus (Segerdell et al. 2013), chicken (Wong et al. 2013), and human (Hunter et al. 2003; Rosse and Mejino 2003; Bard 2012). These cross-references enable connections among diverse biological datasets annotated with terms from anatomy ontologies for other species, thus facilitating integration of mouse developmental data within the broader scientific domain.

## Summary and future directions

The EMAPA is available for download from the OBO Foundry, with descriptive information and other documentation presented in associated wiki pages. As of July 2015, the developmental mouse anatomy ontology contains nearly 6300 terms representing anatomical structures covering the entire lifespan of the mouse. Each EMAPA term is associated with information regarding the stages at which it is present, as well as in the context of relationships with other structures. The ontology includes over 9600 relationships between terms, structured in a multi-parental hierarchical organization, providing a means for aggregation and integration of data described at different levels of anatomical granularity. Furthermore, more than 28,500 EMAPS terms can be derived from the primary EMAPA ontology, enabling direct annotation to mouse anatomy terms at specific stages.

The anatomy ontology for the developing mouse, EMAPA, along with stage-specific EMAPS components, will continue to be expanded and refined according to the requirements of ongoing data curation as well as input from the scientific community at large. Optimally, as for the GUDMAP contributions, this will include comprehensive editing of specific areas of the ontology with domain-specific expert involvement. Editorial procedures have been developed to facilitate efficient response to new term requests, enable coordinated ontology editing by GXD and EMAP curators, quality control, repository access, and public release. Following the principles and guidelines set forth by the OBO Foundry, further efforts are underway to improve the anatomical ontologies. These will include addition of comprehensive textual (i.e., human-readable, natural language) definitions as well as formal (computable, logical) definitions that can be used by automated reasoners, and other forms of metadata relevant to the anatomical entities represented by the ontology.

Future development of the mouse anatomy ontology will also involve extension and refinement of relationships between concepts, including the introduction of other types of relationships. Among the early objectives of the mouse anatomy ontology effort was to eventually provide developmental information including lineage for anatomical structures within the ontology. Toward this goal, we are planning to include “develops from” relationships—e.g., brain develops from future brain (see Fig. 2c); heart develops from primitive heart tube—to enable the representation of relevant developmental lineage, and thus to support the analysis of differentiation and lineage pathways for mouse gene expression, phenotypic, and disease-related data.
